# Temporal-spatial trends in potentially toxic trace element pollution in farmland soil in the major grain-producing regions of China

**DOI:** 10.1038/s41598-019-55278-5

**Published:** 2019-12-19

**Authors:** Erping Shang, Erqi Xu, Hongqi Zhang, Caihong Huang

**Affiliations:** 1 Key Laboratory of Land Surface Pattern and Simulation, Institute of Geographic Sciences and Natural Resources Research, Chinese Academy of Sciences, Beijing, 100101 China; 2 Institute of Remote Sensing and Digital Earth, Chinese Academy of Sciences, Beijing, 100094 China; 3 Aerospace Information Research Institute, Chinese Academy of Sciences, Beijing, 100094 China; 4 University of Chinese Academy of Sciences, Beijing, 100049 China; 5 Chinese Research Academy of Environmental Science, Beijing, 100012 China

**Keywords:** Environmental sciences, Environmental chemistry

## Abstract

Pollution from potentially toxic trace elements (PTEs) is becoming serious and widespread in farmland soils in China, threatening food security and human health. Few large-scale studies systematically analyzed their temporal-spatial trends over vast spatially elaborate sites. The soil health status of the main grain producing areas was first announced based on a total of 3662 spatially elaborate farmland topsoil sites from the 1980s to the 2000s. Nearly 21.5% of sites were polluted, although only slightly. Pollution from the Cd, Ni, Cu, Zn, and Hg was more serious. Pollution was more extensive in the south than in the north. There was an increasing trend in the PTE concentrations, especially Cd with a growth of 21–25%, and in the proportion of mixed pollution at the sites (19.3%), Cd (21.5%), Pb (3.6%), Zn (5.7%), Cu (7.0%), and Hg (3.1%). Furthermore, temporal variations in severe Cd pollution and mixed-level Hg pollution in the north are severer. This study may provide guidance for policymakers regarding the protection and high-risk area of PTE contamination in the soils.

## Introduction

“Potentially toxic trace elements (PTEs)”, which are more inclusive and appropriate than the term “heavy metals^1^” in soils, are an issue of increasingly serious concern because of their persistence and biotoxicity^2–4^. The latter could potentially threaten human health via the food chain^5–9^, especially if these soils are found on arable land and used to grow vegetables and cereals^10–14^. Soil contaminated with PTEs is also becoming an increasingly severe problem because of rapid industrialization and urbanization, and other changes that occurred to agricultural soil in most parts of the world in the last few decades^15–18^. The addition of fertilizers, fungicides, organic amendments, and sewage sludge can have significant detrimental effects^19^ on food safety, ecosystems, and human health^20–22^; these have also been identified as co-factors in many diseases^23^. Numerous recent pollution incidents linked to PTEs (such as “Cadmium rice”) in China^24,25^ have led to increasingly strong concern^25–29^. The Law of the People’s Republic of China on Soil Pollution Prevention and Control will be implemented on January 1, 2019. China has begun a “war^30^” to conquering soil pollution to prevent and control soil heavy metal pollution to protect the safety of agricultural products and human health. How the soil pollution in the main areas, especially the main grain producing areas, is the most concerned issue.

Previous studies focused on the current state of PTE accumulation in the soil in China. Most research on farmland soils was mainly conducted either over small-scale regions such as villages, towns, and counties^31,32^, a selected typical area (i.e., industrial or mining areas)^33,34^, or with limited cultivated soil sampling points^25^. Some studies also focused on other land use types^35–37^. However, these were insufficient to reveal the overall status of PTE pollution in the agricultural regions of China, where the soil is spatially elaborate soil, without some uncertainties. Furthermore, there are limited reviews of soil PTE pollution in the main grain-producing regions of China, which are important to agriculture in the country^38^. An overall evaluation of the main grain-producing regions of China with vast spatially elaborate soils is urgently needed.

However, to date, there has been only limited regional-scale research on temporal trends in PTE contamination in cultivated soils, and little is known about the spatial characteristics. There have also been a few small and medium-scale local studies^39–43^. International researchers have focused on urban soils^44^, mining soils^45^, and industrial soils^46^; less research on cultivated soils. In China, only a few scholars have analyzed the PTEs content in the small and medium-scale scales by obtaining the measured data of soil PTEs in different time periods. For example, Long *et al*.^47^ analyzed the trend of PTEs content in farmland soil in six periods in Shanghai; Lu *et al*.^48^ comprehensively compared the statistical characteristics and annual trends of PTEs content of three different agricultural land use such as orchards, grain fields and vegetable plots in Beijing from 2005 to 2009; Yang *et al*.^49^ analyzed the temporal and spatial variation of PTEs in paddy soils polluted by the mining area along the Hengshi River from 2004 to 2012; Song *et al*.^50^ comparative analysis of PTEs changes in paddy soils in Wenling City, Zhejiang Province in 2006 and 2011. Also, some people used space-time-changing methods or constructed models to analyze trends of soil PTEs content. For example, Hao^51^ analyzed the PTEs in farmland soils in Cixi for 1000 years according to the time series and mass balance theory. Nevertheless, regional temporal trends of PTEs in the soil environment are very important for environmental risk management^52^ as they can help decision-makers better understand target pollutants and thus make more informed environmental management decisions^40^ related to human health. The lack of a long-term monitoring network means that field surveys and retrospective analyses of published investigations of PTE concentrations are valuable methods with which to comprehensively reveal regional-scale temporal trends in PTE concentrations in cultivated soils and that these can provide deeper insights into environmental behaviors of pollutants than individual field surveys can. However, such research on PTE concentrations in farmland soil in the main grain-producing regions of China is not available.

The Five Main Grain-producing Regions (FMGPRs) of China, namely the Sanjiang Plain (SJ), Songnen Plain (SN), Yangtze River Middle and Jianghuai Plains (CJ), Huang-Huai-Hai Plain (HHH), and Sichuan Basin (SC), comprise more than 60% of the total cropland area in China and produce nearly 70% of the food consumed in the country (http://www.zzys.moa.gov.cn). PTE pollution in these regions would pose a threat to the quality and safety of basic and processed agricultural products and human health throughout China and around the world. Therefore, it is necessary to assess the temporal trends of PTEs in the agricultural soils of the FMGPRs. The main objectives of this study were to 1) determine the current status of PTEs in vast, spatially elaborate farmland topsoil over a large scale in the FMGPRs using the single factor index and revised comprehensive pollution index methods, 2) assess their temporal trends, from the 1980s to the 2000s, at the site- and grid-scale, and 3) to provide guidance for the policymakers regarding environmental management and the reduction and restriction of areas of high-risk soils for future food security and human health.

## Material and Methods

### Sample collection and analysis

This study systematically reviewed several studies on soil PTE concentrations in the FMGPRs of China. Of these, the SJ, SN, and HHH are in northern grain-producing regions (NGPRs) while the CJ and SC are in the southern grain-producing regions (SGPRs) (Supplementary Text S1).

**Figure 1:**
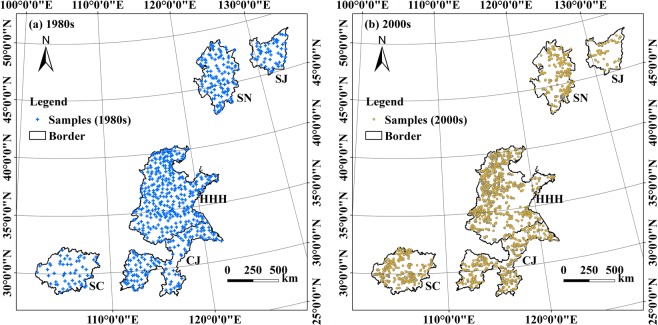
Spatial distribution of the samples in the five major grain-producing regions in China in the 1980s (**a**) and 2000s (**b**).

A total of 3,662 spatially elaborate farmland topsoil samples were collected in the FMGPRs from the 1980s to the 2000s (Fig. 1). Among them, 656 samples for the period 1982–1990 were obtained by digitizing graded point site maps of Cd, Pb, As, Ni, Cu, Zn, Cr, and Hg from “The Atlas of Soil Environmental Background Values in the People’s Republic of China^53^”. We collected another 1,393 topsoil samples from farmland in the FMGPRs from 2013–2014, and a further 1,613 total samples were derived from the 398 papers published in the 2000s (Supplementary Table A1) on the China National Knowledge Infrastructure (CNKI) and Web of Science. The studies reviewed met the following criteria: (1) keywords included “soil,” “heavy metal,” “PTE,” “agriculture,” “farmland,” “China,” and the names of the provinces that make up the FMGPRs, (2) dates between 2000 and 2017, (3) data from field surveys of the targeted heavy metals in farmland topsoil, (4) content and geographical distributions of the target PTEs were obtained directly or through the calculation. Data from humanities and social science journals and soil from industrial and urban land, construction sites, parkland, water bodies, and sediments, were excluded.

We assumed that data from 2000–2017 and 1982–1990 were derived from the same period, termed the “2000s” and “1980s”, respectively. As these sites were found across every province and city in the FMGPRs (except SJ), the analysis is relatively comprehensive and provides a large-scale assessment by combining the data from previous papers.

### Physical analysis

For the samples from the reviewed studies, soils at each sampling site were collected in three or five cores (0–20 cm depth or 0–15 cm depth) using a bamboo spade, stainless steel spade, or plastic scoop and packed into a single kraft bag or plastic packages using random or uniform sampling methods. While 656 soil samples from the 1980s were spread on a plastic sheet via uniform sampling methods. We collected a mixture of five sub-samples (0–20 cm depth) into plastic packages using a plastic scoop. Random sampling methods were used in the areas with a small number of sampling points or no sites, according to the published literature, to fully cover the main cultivated areas in the FMGPRs. In the laboratory, all soil samples were air-dried, ground, and sieved (through 2 mm mesh) before chemical analysis. The pH of a portion of each soil sample was also measured. The remaining soil was then passed through a 100 mesh nylon sieve (150 mesh nylon sieve in the 1980s) to analyze the PTE content.

### Chemical analysis

In all the studies we reviewed and field surveys we conducted, the total concentrations of PTEs in soils were analyzed by digesting the samples usually using a concentrated mixed acid, such as HNO_3_-HClO_4_-HF, or HCl-HNO_3_-HClO_4_-HF, or aqua regia (HNO_3_-HCL) and so on. They are all listed in the international standards and national standards, the PTEs contents in soil by them were compatible with the quality control range. A more detailed description of these procedures can be found in the paper^54^. The soil PTEs were then determined using various methods (i.e., ICP-MS, ICPAES, ICP-OES, CV-AAS, AAS, XFS, or XRF), which are accepted by the scientific community and widely used^40,55^. These methods were applied using strict quality control and assurance procedures. Therefore, the combination of published studies and field surveys were generally valid.

### Quality control and assurance

Quality control and assurance of analytical accuracy were guaranteed for all samples. Among them, geochemical reference materials (i.e., ESS and GSS) were used mainly for data from the reviewed studies and the 1980s, with relative standard deviations (RSDs) below 15% for the eight PTEs in the 1980s and less than 10% in the 2000s. The analytical procedures used to validate the accuracy of the PTE analysis included careful standardization, procedural blank measurements, and spiked and duplicate samples^56^. For the field survey we conducted, we also used GSS as analytical quality control and achieved RSDs below 10% for the eight PTEs. This also indicates that combining the published studies and field surveys were also valid in general.

### Pollution assessment

#### Pollution assessment for individual PTEs

Single pollution indexes can be used to rank contamination levels. The pollution index (*PI*) used herein^57,58^ is defined as follows:

1 $${P}_{i}={C}_{i}/{S}_{i},$$

Where *P*_*i*_ *C*_*i*_, and *S*_*i*_ are the single pollution index, measured value (mg·kg^−1^), and evaluation standard (mg·kg^−1^) of PTE *i* for the given situation. The Grade II values (GB15618–1995) which are different from the soil pH values (Supplementary Table A2) in soils^59^, were used as the standards by which the results of this study were evaluated; this standard is common in China^60^.

The *PI* was divided into five levels to indicate the degree of contamination: clean (0 <*P*_*i*_ ≤ 0.7), sub-clean (0.7 <*P*_*i*_ ≤ 1), slightly polluted (1 <*P*_*i*_ ≤ 2), moderately polluted (2 <*P* _*i*_≤ 3), or severely polluted (*P*_*i*_> 3)^61,62^.

#### Pollution assessment for every site

At each sample site, the soil might have been contaminated with one or more PTEs; therefore, there may be multiple PI values greater than 1. The Nemerow pollution index (*NPI*) was used, in general, to evaluate the comprehensive pollution level of PTE pollution in the soil^63^. However, in addition to emphasizing the influence of the maximum single pollution index, this study also considered the average concentration^64^. This can produce easily generated, biased assessment results in terms of accurately determining whether or not the PTE at a site exceeded the standard; that is, whether or not it was an “excessive site” (ES). For example, the NPI at one site was 0.98 if the eight PTE PI values were 1.2, 0.3, 0.6, 0.7, 0.8, 0.5, 0.54, and 0.62; however, the NPI would show that this site was clean, since the former ignored the excess elements that existed at this site.

To address this issue, we defined the term “excessive site” (ES) as one from which a sample had PTE PI values greater than 1, the slightly lower pollution limit. The pollution level of an ES was determined by the maximum *PI* (MPI). The revised comprehensive pollution index can, therefore, be expressed as follows:

2 $$MPI={P}_{{\rm{site}}}=\,\max ({P}_{i})$$

Where *P*_site_ is the pollution index of the site and *P*_*i*_ is the single pollution index of PTE *i*. We also defined it into five levels: clean (0 <MPI ≤ 0.7), sub-clean (0.7 < MPI ≤ 1), slightly polluted (1 < MPI ≤ 2), moderately polluted (2 < MPI ≤ 3), or severely polluted (MPI > 3). The ES percentage refers to the proportion of total sample sites meeting the ES criteria^60^.

### Statistical analysis and spatial mapping

The data are presented as the mean ± standard deviation (SD). Statistical analysis was performed using SPSS 18.0. The 10^th^, 25^th^, 50^th^, 75^th^, and 90^th^ percentiles, mean, SD, and coefficient of variation (*CV*) of the PTE concentrations was calculated. The normality of the data was evaluated in SPSS, and the standardized skewness and kurtosis values indicate significant departures from normality for all eight PTEs (Table 1). Furthermore, the soil PTE content in the 1980s were classified into eight grades (5%, 10%, 25%, 50%, 75%, 90%, 95%, 100%) (Supplementary Table A3) using the unified numeral grading method. The soil PTE content in the 2000s was also classified using the same system to allow the evaluation of spatio-temporal changes in the PTEs between the 1980s and the 2000s. Meanwhile, the original eight grades were divided into the categories “clean”, “mixed pollution”, and “serious pollution”. Mixed pollution mainly refers to a combination of mild and moderate pollution. Serious pollution is that most of the content of PTEs in the samples is far more than 3 times the standard value. Their concentration range can be seen in Supplementary Tables A3 and A4.

**Table 1:** Summary statistics of the concentrations of PTEs in Chinese farmland soils in the 2000s (mg·kg^−1^).

Properties	Cd	Pb	As	Ni	Cu	Zn	Cr	Hg
Sites	2,784	2,889	2,137	1,664	2,498	2,150	2,510	2,152
10th	0.073	16.34	5.04	19.29	14.46	46.00	35.60	0.025
25th	0.111	20.66	6.95	23.97	19.36	58.74	51.90	0.034
50th	0.159	24.97	8.87	27.76	23.94	71.70	62.30	0.052
75th	0.266	33.85	10.80	32.87	29.67	90.32	72.51	0.090
90th	0.538	50.00	13.17	41.25	40.78	123.59	86.21	0.161
Mean	0.329	29.36	9.74	29.43	26.31	84.43	62.75	0.104
SD	0.691	16.13	10.09	10.84	13.54	70.45	22.63	0.239
Mean*	0.537	30.69	11.62	32.56	28.07	104.92	65.40	0.207
SD*	5.61	27.97	91.14	59.77	32.68	381.29	44.60	3.03
Skewness	24.63	13.23	45.47	18.54	14.78	22.98	11.60	19.55
Kurtosis	696.70	283.06	2089.65	361.57	280.95	612.62	214.61	394.75
CV (%)	930	91	785	183	116	363	68	1144
NES_C	2460	1684	842	1176	1695	1252	1602	1488
NES_P	2354	1556	513	719	1466	1119	1061	1608
BV	0.097	26	11.2	26.9	22.6	74.2	61	0.065

Mean*: mean with abnormal values; SD: standard deviation; SD*: standard deviation with abnormal values; CV: coefficients of variation; NES_C: numbers of sampling sites with concentration exceeded the background values in the whole China; NES_P: numbers of sampling sites with concentration exceeded the background values in their provinces; BV: background values in China. The abnormal values are a non-measurement error. That is also a true reflection of the pollution status of PTEs in the soil, indicating that the soil has a very high level of PTEs.

The spatial distributions of the PTEs in the 1980s and 2000s were mapped using the inverse distance weighted (IDW) method and at the point scale in ArcGIS 10.1. The distance between the interpolation point and the sample point was used to weight the average, such that shorter distances conferred a greater weight. The predicted value was equal to the measured value at the sample point, and the original maximum and minimum values were not changed. In addition, the high spatial heterogeneity in the agricultural soils, farming methods, pollution sources, and other factors induces high spatial heterogeneity in the soil type of the FMGPRs. This introduces uncertainties into any spatial distribution maps produced via spatial interpolation methods. Therefore, we also focus on an overall analysis of the percentages of sites with the various pollution levels and different point scale PTE pollution levels within the total study region during the 1980s and 2000s to better explore trends in PTEs levels.

## Results and Discussion

### PTE concentrations in soils

Descriptive statistics for PTE concentrations in soils from the FMGPRs in China are presented with the relevant geochemical background values (BVs)^65^ in Table 1. The arithmetic means of Cd (0.329 ± 0.691), Pb (29.36 ± 16.13), As (9.74 ± 10.09), Ni (29.34 ± 10.84), Cu (26.31 ± 13.54), Zn (84.43 ± 70.45), Cr (62.75 ± 22.63), and Hg (0.104 ± 0.239) (all units in mg·kg^−1^) are higher than the corresponding local BVs. The median concentrations of Cd (0.159 versus BV limit of 0.097), Ni (27.76 versus BV limit of 26.9), Cu (23.94 versus BV limit of 22.6), and Cr (62.30 versus BV limit of 61) are higher than the background values in China (Table 1) (all units in mg·kg^−1^). This suggests that in at least half of the farmland soil samples, these four PTEs come from anthropogenic sources as well as from the parent rock. On the other hand, the median concentrations of Pb, As, Zn, and Hg are slightly below their BV limits. Approximately 84.6% of the Cd samples and 74.7% of the Hg samples surpass their provincial BVs, followed by Cu (58.7%), Pb (53.9%), and Zn (52.0%); 24% to 43% of the As, Cr, and Zn samples.

Table 1 also shows significant heterogeneity in the spatial distribution of eight PTEs, which is reflected in the high SDs and CVs (from 68% to 1144%). Samples are considered highly variable and dispersed when the CV > 36%^66^. The kurtosis values of all eight PTEs are greater than zero, demonstrating that the distributions of the concentrations of these elements in the samples are narrower than a normal distribution. Furthermore, kurtosis and skewness values for typical PTEs (Cd, As, Zn, and Hg) are higher than those for other PTEs indicating the existence of highly contaminated areas.

### Distribution and pollution assessment

#### Comparative analysis of excessive site (ES) percentages

Of the 3006 soil samples analyzed in the 2000s, 21.5% exceeded the GB15618–1995 environmental quality standard^59^, and 20.1% exceeded the risk screening value (GB 15618–2018). They are slightly higher than the ES percentage (19.4% of the cultivated land) described in the National Bulletin on Soil Pollution Status in 2014^60^. It can be seen that the results of the new standard (GB 15618–2018) are slightly lower than the old standard (GB15618–1995), but the difference is small. And because GB15618–1995 standard can be better compared with the published literature of the peers, especially the officially published authoritative reports^60^, which all used GB15618–1995, and the main evaluation criteria are also based on GB15618–1995. The dominant ES pollution level in the samples was “Slightly polluted (14.0%); the others were “moderately polluted” (2.5%) and “severely polluted” (5.0%) (Table 2).

**Table 2:** Percentages of potentially toxic trace elements in farmland soils, based on class distributions used in the pollution assessment and percentage of excessively polluted sites in the 2000s.

Regions	Sites	Numbers of ES	Percentage of ES (%)	Slight pollution (%)	Moderate pollution (%)	Severe pollution (%)	Percentage of exceeds the risk screening value (GB 15618–2018) (%)
SJ	60	1	1.7	0.0	1.7	0.0	1.7
SN	353	33	9.4	2	4	3.4	10.0
CJ	731	224	30.6	21.6	1.9	7.1	30.4
HHH	1350	165	12.2	5.8	1.3	5.1	11.1
SC	512	223	43.6	34.6	5.5	3.5	33.6
Whole	3006	646	21.5	14.0	2.5	5.0	20.1

**Figure 2:**
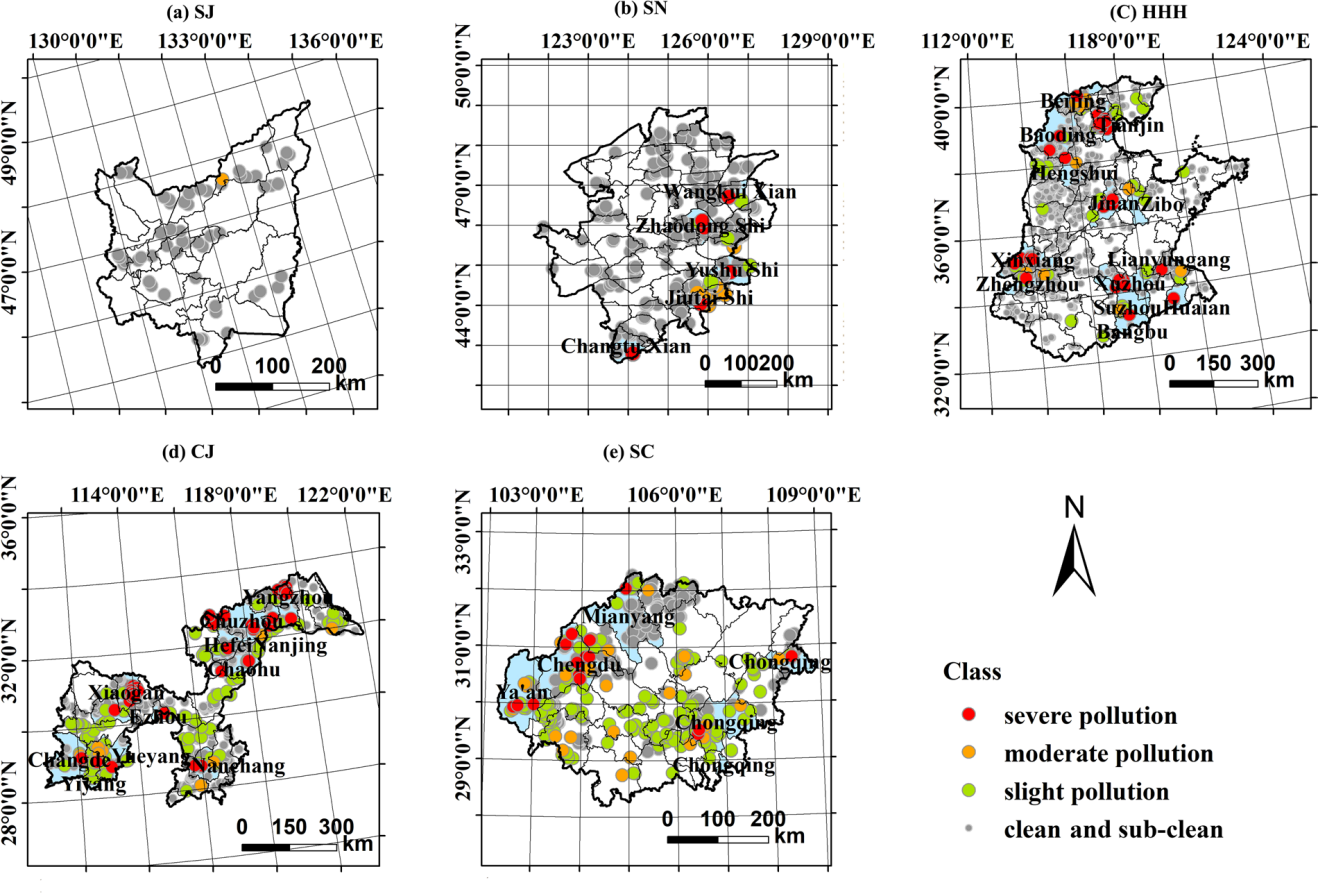
Spatial distribution of the pollution levels of potentially toxic trace elements in the farmland soils in the five major grain-producing regions in China. (**a**) SJ; (**b**) SN; (**c**) HHH; (**d**) CJ; (**e**) SC.

The soil PTE pollution is heavier in the SGPRs than in the NGPRs (Fig. 2, Table 2). The SC region features the highest ES percentages (43.6%), followed by CJ (30.6%), HHH (12.2%), SN (9.4%), and SJ (1.7%). Moreover, the “slightly polluted” ES percentages are larger in the SC (34.6%) and CJ (21.6%) than in the other regions, each of which has less than 6%.

The CJ region had the most severely polluted samples (7.1%). These were collected in cities in the north (i.e., Yangzhou, Chuzhou, Hefei, and Huaian), west and southwest (Yiyang, Changde, and Xiaogan), and southwest along the border of Nanchang. The HHH possessed the second-highest severe pollution ES percentages (5.1%); these samples were collected near northern, central, and southeastern cities including Beijing, Tianjin, Baoding, Hengshui, Jinan, Xinxiang, Zhengzhou, Jiaozuo, Xuzhou, Lianyungang, Bengbu, Huaian, and Nanjing.

The SC and SN have severely polluted ES percentages of 3.5% and 3.4%, respectively. The ESs in the SC are primarily distributed on its western margins, including Chengdu, Deyang, and Ya’An; the northern city of Mianyang; and the southeastern city of Chongqing. The severely polluted ESs in the SN are located largely in the east and south, including Wangkui County, Zhaodong, Yushu, Haerbin, Jiutai, and Changtu. There were no severely polluted ESs in the SJ).

Overall, the highest ES percentages were found in the SC. The highest ES percentages, indicating severe pollution, were found in the CJ region followed by the HHH. Most samples from the SJ and SN can be considered clean.

#### Comparative analysis of major PTEs using the Pollution Index

The *PI* values of the eight PTEs vary widely, ranging from clean to severely contaminated (Supplementary Fig. A1, Supplementary Table A5). The major pollutants are Cd, Ni, Cu, Zn, and Hg; 17.4%, 8.4%, 4.0%, 2.8%, and 2.6% of the *PI* values of these pollutants are higher than 1; less than 1% of other PTEs are >1. PTE contamination of cultivated land is more concentrated in the SGPRs. Moreover, slightly polluted areas account for 60% of the Cd, Zn, and Hg measurements; 85% of the Cu samples; and 92% of the Ni samples.

**Figure 3:**
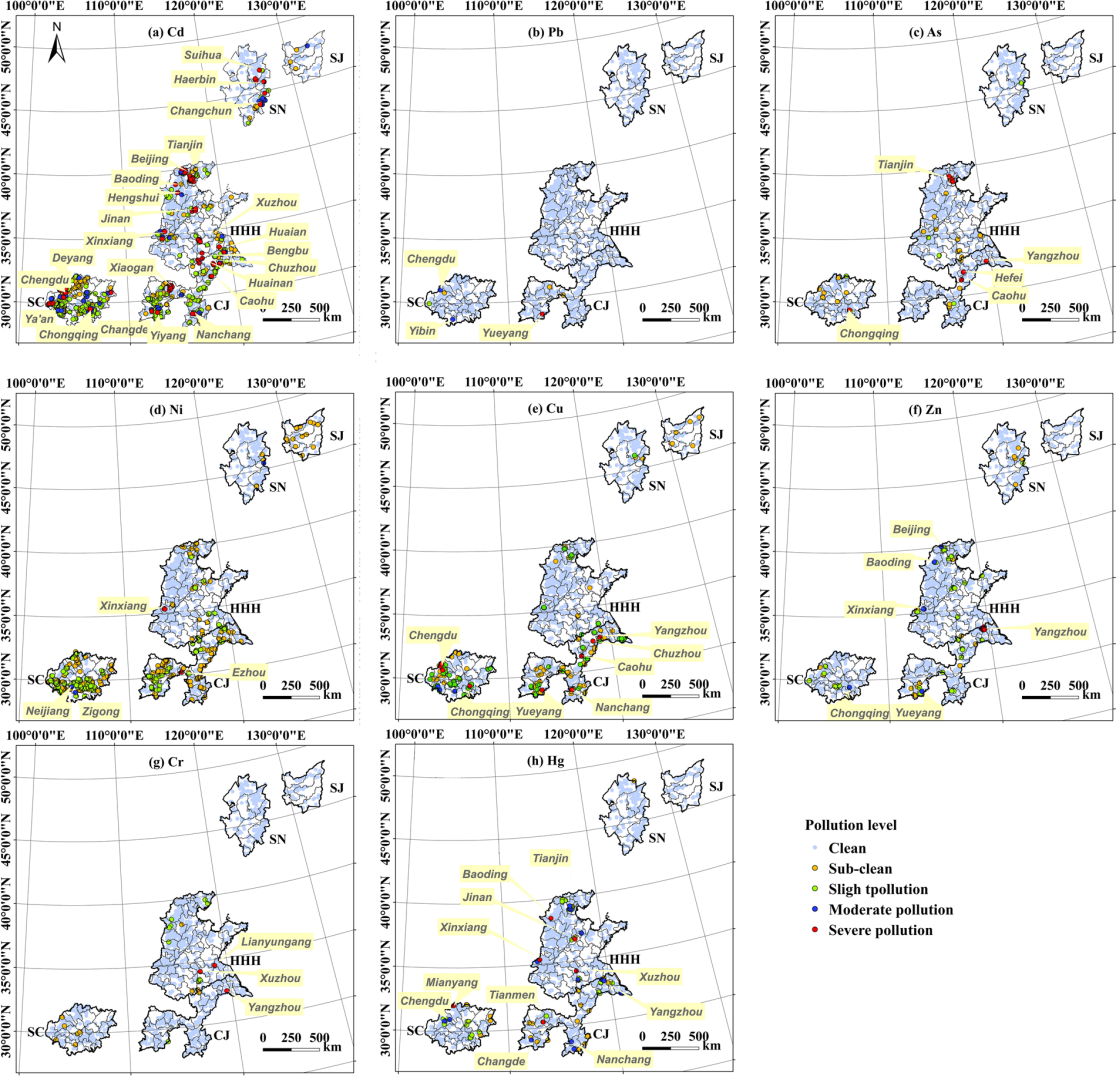
Potentially toxic trace element pollution levels in the five major grain-producing regions in China. (**a**) Cd, (**b**) Pb, (**c**) As, (**d**) Ni, (**e**) Cu, (**f**) Zn, (**g**) Cr, (**h**) Hg.

The agricultural soils in the FMGPRs are contaminated by Cd; it was the dominant pollutant in all regions, with *PI* values above the slightly polluted level in 17.4% of the samples. The percentage of Cd *PI* values above the slightly polluted level varies widely, ranging from 1.7% in the SJ to 34.9% in the SC. The SC features the highest proportion of slightly polluted sites (27.1%); these were located in the western cities of Chengdu, Deyang, and Ya’An. Meanwhile, the eastern city of Chongqing had the highest number of severely polluted sites (2.9%) (Fig. 3). In the CJ, which had the highest Cd *PI* values, approximately 21.9% of the samples had Cd *PI* values greater than 1. The sites with severe Cd pollution levels (5.2%) were located in the northern cities of Yangzhou, Nanjing, Chuzhou, and Huaian; the western cities of Xiaogan and Changde; Yiyang in the southwest; and Nanchang in the south. The HHH featured the third-highest percentage of Cd pollution (10.8%). Samples showing severe pollution came predominantly from Nanjing, Yangzhou, Xinxiang, Jinan, Tianjin, Hengshui, Beijing, Xuzhou, Huaian, Zhenjiang, and Bengbu. The SN featured a Cd pollution percentage of 9.8%. Severely polluted samples (3.8%) were distributed in the northeastern and southern SN, including Wangkui County, Harbin, and Zhaodong.

Nickel and Cu pollution are not as extensive as that of Cd; 8.4% of the Ni samples and 4.0% of the Cu samples indicate more than slight pollution levels. Less than 3% of Ni samples and 1% of Cu samples in the NGPRs were more than slightly polluted, and these figures are above 16% for Ni and 8% for Cu in the SGPRs. However, there were extreme Ni values in the HHH; this was unlike Cu, where the extreme values were located in the CJ and SC. In the CJ and SC, 16–31% of the Ni samples and 8–9% of the Cu samples featured more than slight pollution; this number was less than 3% for Ni and Cu in the other study regions. However, Ni and Cu contamination were not severe in general. Less than 8% of the polluted samples were moderately polluted, and less than 14% were severely polluted. The distributions of Ni and Cu in the SC were distinctly different from those in the CJ. In the CJ, the more than slightly polluted samples came chiefly from the north and southwest. However, small pockets of severe Ni pollution (0.3%) were found in the central city of Ezhou. In a line of cities from southwest to northeast, 1.0% of sites featured Cu pollution classified severely polluted above. In the SC, Ni, and Cu pollution occurred mainly in the western and southern areas. A small number of moderately polluted samples were found in Neijiang and Zigong, while severe pollution was recorded in samples from Chengdu and Chongqing, which surround industrial and mining regions.

In the FMGPRs, 2.8% and 2.6% of the samples of Zn and Hg, respectively, were more than slightly polluted. The highest Zn and Hg *PI* values were both located in the CJ, which was sampled most heavily. Accordingly, 4.6% of the Zn samples and 3.0% of the Hg samples from this region featured more than slight pollution. In the HHH and SC, 2–3% of the soil samples had an excess of Zn and Hg were; this value was less than 0.5% in the SN and SJ. Slightly polluted samples were dominant. In the CJ region, the samples with the highest Zn contamination were found near cities in the southwest and northeast, particularly in Yangzhou and Yueyang. Conversely, Hg pollution occurred largely in the northern city of Yangzhou, the western city of Tianmen, and the southwestern city of Changde. In the HHH region, samples with Zn pollution were chiefly from near the northern cities, along a line from northwest to southeast, notably including Beijing, Baoding, and Xinxiang, which had the highest levels of Zn contamination. Small pockets of severe Hg pollution occurred in Baoding, Xinxiang, Ji’nan, and Xuzhou. In the SC, Zn pollution was concentrated in the northwestern and south-central cities, especially Chongqing. Hg pollution tended to be higher in the northwestern and central cities, especially Mianyang and Chengdu in the former regions.

### Temporal and spatial trends

#### Trends of concentrations of PTEs in soils

**Figure 4:**
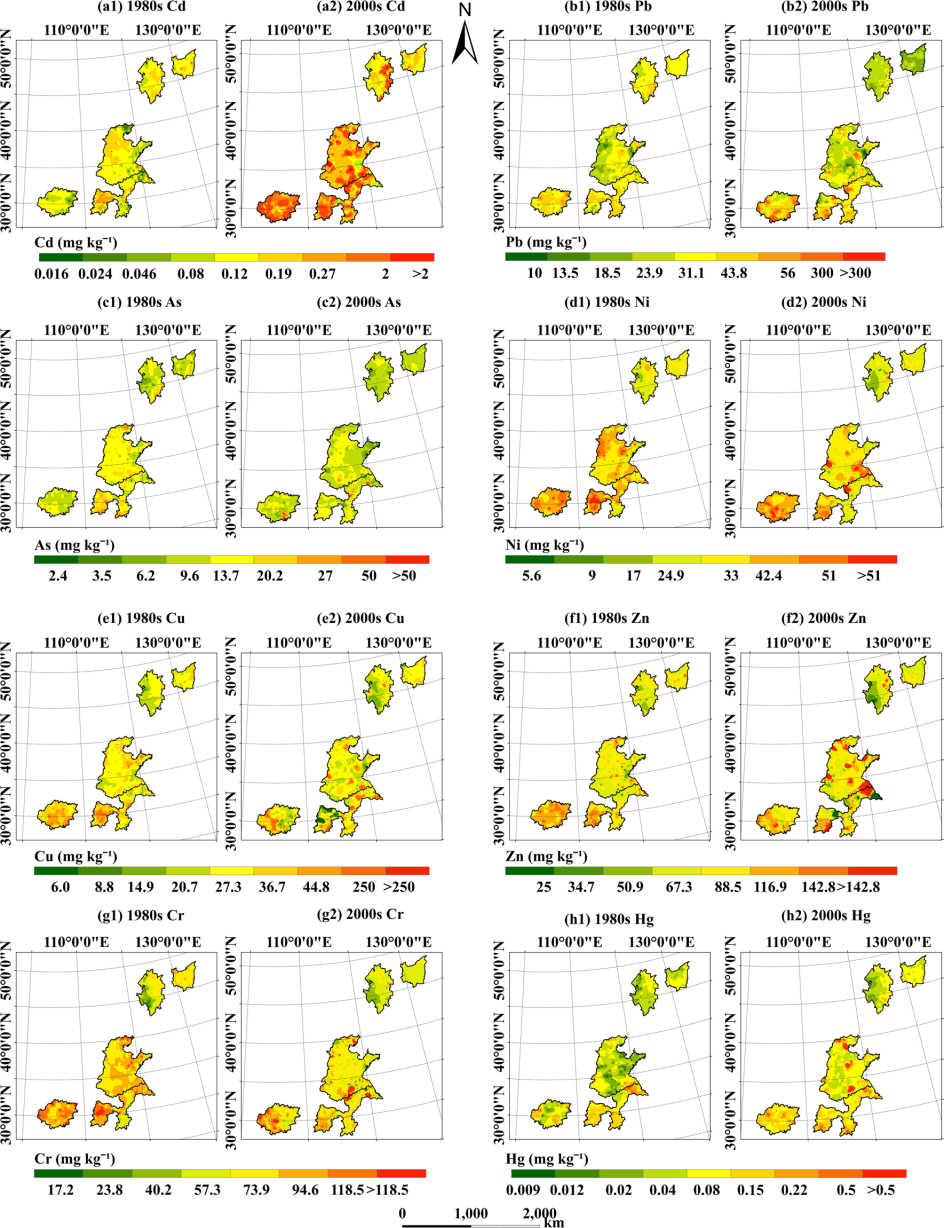
Trends in the concentrations of potentially toxic trace elements in the five major grain-producing regions in China from the 1980s to 2000s. (**a1**) 1980sCd, (**a2**) 2000sCd, (**b1**) 1980sPb, (**b2**) 2000sPb, (**c1**) 1980s As, (**c2**) 2000s As, (**d1**) 1980s Ni, (**d2**) 2000s Ni, (**e1**) 1980s Cu, (**e2**) 2000s Cu, (**f1**) 1980s Zn, (**f2**) 2000s Zn, (**g1**) 1980s Cr, (**g2**) 2000s Cr, (**h1**) 1980s Hg, (**h2**) 2000s Hg.

The PTE concentrations in cultivated soils in the FMGPRs increased, in general, between the 1980s and the 2000s (Fig. 4). The proportion of low PTE concentrations decreased while the proportion of high PTE concentrations is increasing.

The concentrations of Cd, Pb, Zn, Cu, and Hg have increased; for Cd, the difference was severe. The proportions of these elements in grades 8 and 9 increased by 25.0%, 4.2%, 6.1%, 3.6%, and 3.1%, respectively. In the 1980s, 55.5% of the study area contained 0.08–0.12 mg·kg^−1^ of Cd, which could be categorized as “clean.” However, in the 2000s, most measurements showed Cd contents between 0.12–0.19 mg·kg^−1^ and 0.27–2 mg·kg^−1^, and the proportion of Cd in the mixed pollution range (0.27–2 mg·kg^−1^) increased from 0.5% to 24.5%. The changes in Hg content are similar to those in Cd. Hg content increased from being predominantly within the 0.02–0.04 mg·kg^−1^ range in the 1980s to lying between 0.08–0.15 mg·kg^−1^ in the 2000s. The increases in Pb, Zn, and Cu were relatively low, the areas affected by grade 8 and 9 pollution increased somewhat (3–6%); the concentrations of As, Ni, and Cr have increased by less than 1% (Fig. 4).

There are also significant regional differences in the high concentrations (grade 8–9) of Cd, Pb, Zn, Cu, and Hg. The increase in the area of high concentration of Cd (8–9) is the largest in the SC (34.4%), followed by CJ (21.7%) and HHH (17.7%). The changes in Pb and Cu are similar to those in Cd and increased more in the SGPRs, which increased by 5.8%-13.0% for Pb, 3.0%-9.3% for Cu, while1.3% for Pb, 3.0 for Cu in HHH. However, the increase of Hg showed the opposite trend, and the highest growth was in HHH (6.4%), and less than 1.5% in SGPRs.

#### Trends at the point-scale ES percentages in different regions

**Figure 5:**
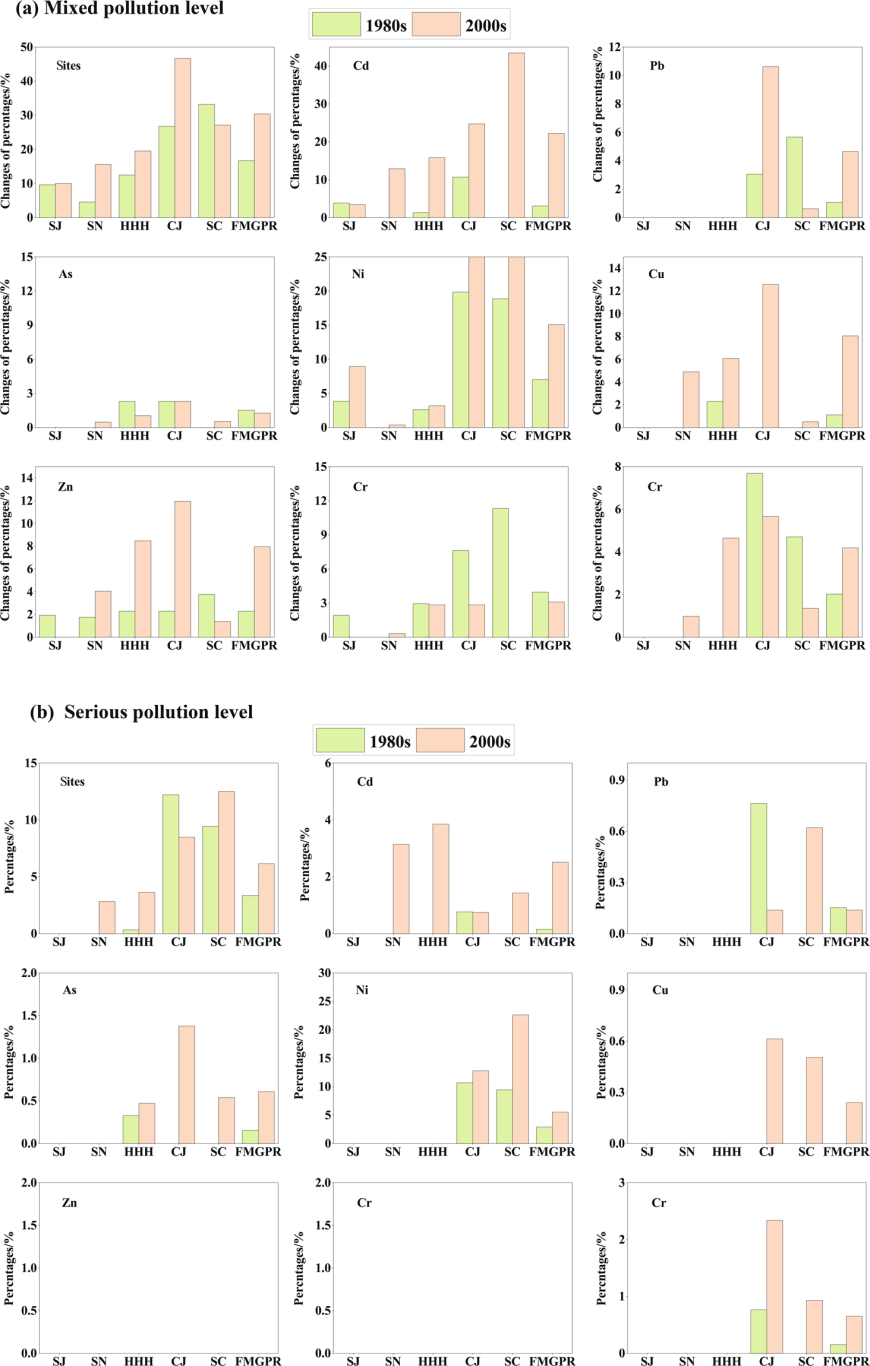
Changes in the percentages of the total sampled sites at the different pollution level in different regions from the 1980s to the 2000s.

For the pollution level, the proportion of sites in the FMGPRs with higher than mixed pollution levels increased from 13.9% to 33.2% from the 1980s to the 2000s; this was an overall growth of 19.3% (Fig. 5, Figure A2). Of these increases, the proportions of samples showing mixed and serious pollution levels increased by 16.5% and 2.8%, respectively. The increases in ES percentage differed by region. In the SJ, PTE pollution tended to vary within the “clean” range over time. However, there was marked variation in the mixed and severe pollution levels in the remaining four regions.

The percentage of ESs above the mixed pollution level increased the most in the SC (29.4%); it was 1.90 times the growth in the SN, 2.21 times that in the CJ, and 2.25 times that in the HHH. Severe pollution levels saw only ~3% growth in all regions, while the percentage of ESs at the mixed pollution level increased by more than 13% in all regions except the SJ.

The mixed pollution level increased more in the SGPRs (Fig. 5) than in the NGPRs; the highest increase was in the SC, at 26.3% this was 1.9 times the growth in the SN, 1.6 times that in the CJ, 2.5 times that in the HHH, and 68.4 times that in the SJ. Seriously polluted samples increased by only 3% in the SN, HHH, and SC, and no severe pollution was observed in the SJ.

The proportion of clean samples decreased by about 19%. This decrease was the highest in the SC (29.4%), followed by 16.7% in the SN and 13% in the HHH and CJ.

#### Trends in point-scale individual PTEs in different regions

The increases in Cd, Pb, Zn, Cu, and Hg PTE contamination was relatively serious; the number of sites contaminated above the mixed pollution level increased by 21.5%, 3.6%, 5.7%. 7.0%, and 3.1%, respectively, for these pollutants (Fig. 5, Figure A3). Furthermore, there were more temporal variations in severe Cd pollution and mixed Hg pollution in the NGPRs than in the SGPRs; the opposite trends were observed for the percentages of Ni, Cu, Zn, and Pb samples at the mixed pollution level.

Cd concentrations have increased markedly. Samples at or above the mixed pollution level increased by 21.5% overall; mixed and severely polluted samples have increased by 19.2% and 2.4%, respectively. At the mixed pollution level, the highest increases were observed in the SC (43.5%), followed by the HHH (14.5%), CJ (14.0%), SN (12.9%), and SJ (−0.4%). However, the increases in measurements of severe pollution were higher in the NGPRs (above 3%) than in the SGPRs (less than 1%).

The proportions of Hg and Pb above the mixed pollution level increased by ~3%. The HHH featured the largest increase in mixed-level Hg contamination, followed by the SN (1.0%). However, the increases in severe pollution were higher in the SC (0.9%) and CJ (1.6%) than in the NGPRs (0). Increases in Pb were observed only in the SGPRs (7%).

There was a greater variability in the Ni, Cu, and Zn samples from the SGPRs that had with concentrations higher than the mixed pollution level compared to those from the NGPRs. The increases in Ni measurements above the mixed pollution level were higher in the SC and CJ (38.6% and 6.4%) than in the NGPRs (less than 5.0%). Cu increased most in the SGPRs, with a growth of 12.0%, but increased less than 5.0% in the NGPRs. At the mixed pollution level, Zn increased most (by 9.7%) in the CJ, followed by the HHH (6.2%) and other regions (where increases were less than 3.0%). There were no variations in the number of severe pollution samples from the FMGPRs; serious Zn pollution also remained unchanged in all regions.

The variations in As and Cr were slightly higher in the SGPRs than in the NGPRs. The percentages of seriously polluted As samples increased by 0.5–1.4% in the SGPRs but only by 0–0.2% in the NGPRs. However, the SC, CJ, and HHH featured similar variations (1.4%) in mixed-level pollution. Cr contamination decreased most (−4.8% overall) in the CJ, followed by the NGPRs (−1.9%).

Overall, the increases in severe Cd pollution and mixed-level Hg pollution were larger in the NGPRs than in the SGPRs. The variations in Pb, Cu, and Ni at the mixed pollution level were larger in the SGPRs than in the NGPRs. The SC showed the largest increases in Cd, As, and Ni at the mixed and severe pollution levels. Meanwhile, the CJ featured the largest increases in Pb, Cu, and Zn; Pb, Hg, Ni, Zn, and Cu increased by 3.0–8.1% and Cd increased most, by 21.5%.

### Comparison with the other studies of China

This study found that 21.5% of the agricultural soils in the FMGPRs were excessively polluted; this figure is slightly higher than the results of nationwide surveys conducted in 2014, which found that 19.4% of agricultural soils were contaminated with PTEs^35,60^. The proportions of slight, moderate, and severe pollution found in this study were 14.0%, 2.5%, and 5.0%; these are similar to those found in the national report (13.3%, 2.8%, and 2.9%)^60^. These slight differences may have arisen from the fact that the official sampling method was uniform sampling, while the sampling points in this study were randomly distributed and some of the data from the literature may have contained publication biases such as focusing on soils surrounding mining or industrial areas. This could have increased the overall levels of elevated PTE measured in the FMGPRs. If the “Soil Environment Quality Soil Pollution Risk Control Standards” (GB 15618–2018) is used as the basis for assessment, the PTEs’ content of cultivated soil in the five major grain-producing areas exceeds the risk screening value by 20.1%. The risk of PTEs pollution in cultivated soils in the southern main producing areas (about 30.4–33.6%) is heavier than that in the north (1.7–11.1%). This trend is also consistent with the old standards and nationwide surveys. At the same time, in the future, we will also choose a variety of risk indicators from the perspective of risk, such as “Soil Environment Quality Soil Pollution Risk Management and Control Standards (Trial)” (GB 15618–2018), potential ecological risk index, health risk index, etc, to systematically assess soil PTEs pollution risk levels.

Both our research and the national report show clear increases in the total concentrations of several PTEs in agricultural soil from the 1980s to the present. The increasing PTE concentrations trends in cultivated soils from the FMGPRs, determined using the IDW method, were consistent with those found using the point-scale method, indicating that our results are relatively reliable. Taking the most severe pollutant, Cd, as an example, IDW interpolation shows that the proportion of soil Cd concentrations above 0.27 mg·kg^−1^ increased from 0.6% to 25.5% (nearly 25%); this is similar to the point-scale results, which show an increase from 3.0% to 24.6% (nearly 21%) in the FMGPRs. After mastering the pollution distribution of PTEs, we may pay more attention to the input (contamination source) and output (soil-crop-human body) of PTEs in soil in the future, such as the factors affecting the distribution of PTEs, the locations of emission sources, soil-crop-human risk.

### Comparison with global results

The PTE soil pollution in Chinese farmland does not appear to be the most serious case globally. Although about 20% of the samples exceed the GB15618–1995 environmental quality standard, it should be noted that Chinese soil standards are quite strict. Chinese pollution thresholds are lower than many soil quality standards worldwide, which tend to increase the proportion and severity of soils considered contaminated. This can be illustrated by the Cd data; the soil limits in China are 0.3 mg·kg^−1^ (pH < 7.5) and 0.6 mg·kg^−1^ (pH > 7.5), which are similar to those in Denmark and Finland (0.3 mg·kg^−1^), but lower than in countries that adopted thresholds of 0.4 to 5 mg·kg^−1^^67^. In the UK and Taiwan, soil Cd measurements are 6.7 and 15.7 times the Chinese levels, respectively.

**Figure 6:**
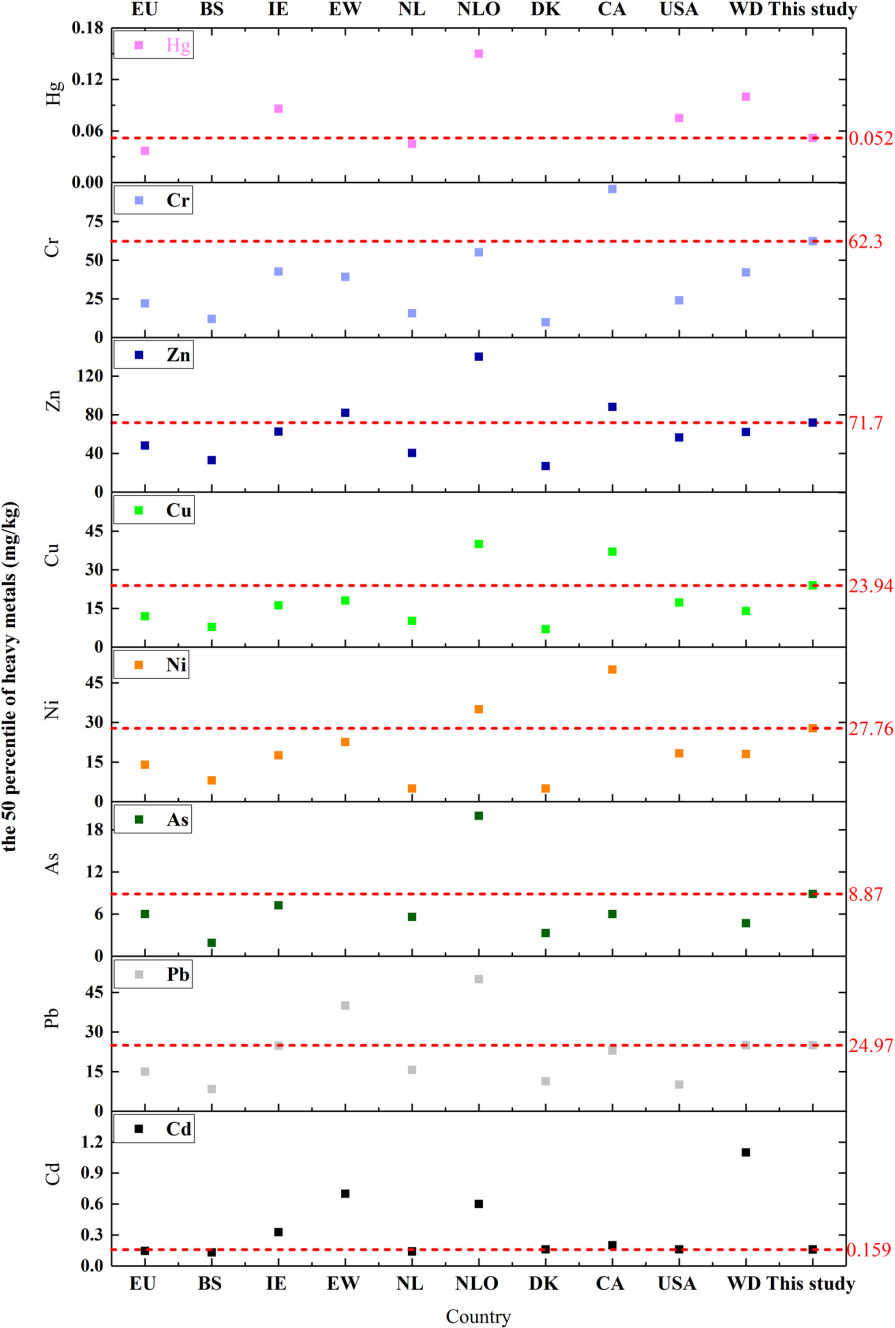
Comparison of the median potentially toxic trace element content in the soil around the world. The red line shows the median PTE contents examined in this study. Abbreviations: EU: Europe^70^; BS: Baltic states^75^; IE: Ireland^73^; EW: England^74^; NL: Netherlands; NLO: Netherlands official^68^; DK: Denmark^71^, CA: California^72^; WD: World.

In addition, a comparison of median PTE values from different countries (Fig. 6) shows that the PTE concentrations in cultivated soils in China are moderate at the international level. Moreover, the concentrations of the eight PTEs studied are significantly lower than those in England and the Netherlands^68,69^.

The median Cd concentration in this study was 0.16 mg·kg-1, similar to those in Europe^6,70^ or other European countries (i.e., Denmark^71^), the United States^72^, and lower than those in the rest of the world^73^. The median Pb concentration was similar to that in the rest of the world and Ireland (both were ~24.97 mg·kg^−1^)^73^, slightly lower than those in England^74^ and the Netherlands^68^, and slightly higher than those in other parts of Europe, the United States^70,72^, and the Baltic Sea^71,75^. The Hg content measured in this study is also significantly lower than the median values for Ireland^73^ and the United States^72^. Ni, Cu, Zn, and Cr concentrations are significantly lower than those in California^76^, but slightly higher than those in the Baltic Sea^75^ and Denmark^71^.

### Study limitations

The data taken from the published literature may contain certain publication biases including the using following: (1) a greater number of samples from heavily contaminated areas than from less-contaminated areas, which may have led to higher proportions of severe pollution, (2) sites that were not uniformly distributed, which may not accurately represent the complete soil PTEs pollution scenario, (3) data from different sources and time periods, which may have introduced some uncertainty to data consistently of a good enough quality to be integrated. In this study, we used the criteria described in Section Chemical Analysis and Section Quality Control and Assurance to reduce errors.

## Conclusions

The concentrations and pollution level of PTEs in agricultural soils in the FMGPRs were determined. Our results, which indicate that about 20% of soil sites are contaminated, agree well with those from the Chinese Ministry of Environmental Protection National Bulletin on Soil Pollution Status Report and provides more spatial data and information on spatiotemporal changes. Agricultural soil PTE pollution is more extensive in the SGPRs than in the NGPRs. We discuss PTE concentration trends from the 1980s to 2000s using the IDW and point-scale methods; both showed increasing trends in contaminated soil (with an increase of 19% over the study period), especially in Cd pollution which had a growth of 21–25%. The SGPRs and HHH Plain are the key target areas for soil pollution control, which may provide some guidance on the prevention of PTE contamination and the protection of soils in high-risk areas in China in the future.

## Supplementary information

Supplementary material

## Data Availability

All data generated or analyzed during this study are included in this published article (and its Supplementary Information files).
