# Supplementary material for: Temporal-spatial trends in potentially toxic trace element pollution in farmland soil in the major grain-producing regions of China
Source: Sci Rep. 2019 Dec 19;9:19463. doi: 10.1038/s41598-019-55278-5 (PMC6923405; doi:10.1038/s41598-019-55278-5)
Supplement: Supplementary file 1 — Supplementary material [file 41598_2019_55278_MOESM1_ESM.pdf]

Supplementary material

Temporal-spatial trends in potentially toxic trace element pollution in farmland soil in the major grain-producing regions of China

Erping Shang [1,2,3,4], Erqi Xu [1,*], Hongqi Zhang [1,*] & Caihong Huang [5]

[1] Key Laboratory of Land Surface Pattern and Simulation, Institute of Geographic Sciences and Natural Resources Research, Chinese Academy of Sciences, Beijing 100101, China; shangep.15b@igsnrr.ac.cn (E.S.)
[2] Institute of Remote Sensing and Digital Earth, Chinese Academy of Sciences, Beijing 100094, China
[3] Aerospace Information Research Institute, Chinese Academy of Sciences, Beijing 100094, China
[4] University of Chinese Academy of Sciences, Beijing 100049, China
[5] Chinese Research Academy of Environmental Science, Beijing 100012, China; huangch@craes.org.cn (C.H.).

***** Correspondence: xueq@igsnrr.ac.cn; Tel.: +86-134-2622-3789
zhanghq@igsnrr.ac.cn; Tel.: +86-136-2116-4004

1 Text
5 Tables
3 Figures

Text 1 Study area

Table A1 Final numbers of sample sites obtained in the five major grain-producing regions of China in the 2000s
Table A2 Chinese Soil Environmental Quality Standards (Total Concentration in mg kg^{-1})
Table A3 Historical data on potentially toxic trace element content grades (mg kg^{-1})
Table A4 The pollution level of the content of the various potentially toxic trace element grades
Table A5 Percentages of class distributions, based on the pollution index (n = 3006), of the assessments of potentially toxic trace element pollution in farmland soils

Fig. A1 The pollution index for potentially toxic trace elements (PTEs) in farmland soils in the five major grain-producing regions of China.
Fig. A2 Trends in the concentrations of potentially toxic trace elements in the cultivated lands of the five major grain-producing regions in China from the 1980s to 2000s
Fig. A3 A comparison map of the spatial distribution of different potentially toxic trace element pollution levels in the cultivated lands of the five major grain-producing regions in China in the two decades (the 1980s and 2000s) at point-scale.

Text 1 Study area

The five major grain-producing regions (FMGPRs) in China (SJ, SN, CJ, HHH, and SC) include the 12 main grain-producing provinces across the country, all of which possess good conditions for agricultural production. According to statistics from the Chinese Farming Information Network (http://www.zzys.moa.gov.cn), the crop planting area in the FMGPRs constitute 69% of the total cropland area in China and about 77%, 73%, and 69%, respectively, of the total planted areas of the main food crops rice, wheat, and maize. The main goal of these FMGPRs is to improve food production. Therefore, the concentrations of PTEs in the FMGPRs directly affect national food security.

The SJ, SN, and HHH are part of the northern grain-producing regions (NGPRs), while the CJ and SC belong to the southern grain-producing regions (SGPRs). There is about 52,000 km^2 of arable land in SJ, most of which consists of middle- and low-yield land that faces frequent floods and droughts. The main crops are corn, rice, and soybeans. The SN is a famous black soil belt in China with an area of 118,000 km^2; it comprises the national corn and rice belt. Organic matter has decreased in the black soil of the SN, and soil salinization has become a prominent issue. The HHH possesses a cultivated land area of about 250,000 km^2 and is the main area for winter wheat production. As the soil becomes shallower and the soil physical properties are degraded, agricultural water becomes extremely scarce, increasing the proportion of middle- and low-yield fields. The CJ contains 72,600 km^2 of arable land area and is the main rice production area. Urbanization has been rapid in this area and soil acidification is intensifying. The SC has a cultivated land area of 118,000 km^2 and is also known for rice production; the soil consists primarily of paddy, purple, yellow, and yellow-brown soil. The high-quality arable land in the SC includes large construction areas, including for industrial and mining, and soil erosion is heavy on the sloped farmland.

Table A1 Final numbers of sample sites obtained in the five major grain-producing regions of China in the 2000s

Regions	Cd	Pb	As	Ni	Cu	Zn	Cr	Hg
SJ (n=60)	58	60	58	56	57	55	59	59
SN (n=353)	318	334	215	272	306	296	304	203
CJ (n=731)	672	725	436	328	652	519	562	428
HHH (n=1350)	1246	1287	1058	787	1087	980	1196	1032
SC (n=512)	490	483	370	221	396	300	389	430
FMGPR (n=3006)	2784	2889	2137	1664	2498	2150	2510	2152

n: numbers of samples; SJ: Sanjiang Plain; SN: Songnen Plain; CJ: Yangtze River Middle Plain and Jianghuai Plain; HHH: the Huang-Huai-Hai Plain; SC: Sichuan Basin; FMGPR: Five major grain-producing regions.

Table A2 Chinese Soil Environmental Quality Standards (Total Concentration in mg kg^{-1})

PTEs		pH		
		<6.5	6.5～7.5	>7.5
Cd		0.3	0.3	0.6
Pb		250	300	350
As	Paddy fields	30	25	20
	dry land	40	30	25
Ni		40	50	60
Cu		50	100	100
Zn		200	250	300
Cr	Paddy fields	250	300	350
	dry land	150	200	250
Hg		0.3	0.5	1

Table A3 Historical data on potentially toxic trace element content grades (mg kg^{-1})

Grade	Cd	Pb	As	Ni	Cu	Zn	Cr	Hg
1	0-0.016	0-10	0-2.4	0-5.6	0-6.0	0-25	0-17.2	0-0.009
2	0.016-0.024	10-13.5	2.4-3.5	5.6-9	6.0-8.8	25-34.7	17.2-23.8	0.009-0.012
3	0.024-0.046	13.5-18.5	3.5-6.2	9-17	8.8-14.9	34.7-50.9	23.8-40.2	0.012-0.02
4	0.046-0.08	18.5-23.9	6.2-9.6	17-24.9	14.9-20.7	50.9-67.3	40.2-57.3	0.02-0.04
5	0.08-0.12	23.9-31.1	9.6-13.7	24.9-33	20.7-27.3	67.3-88.5	57.3-73.9	0.04-0.08
6	0.12-0.19	31.1-43.8	13.7-20.2	33-42.4	27.3-36.7	88.5-116.9	73.9-94.6	0.08-0.15
7	0.19-0.27	43.8-56	20.2-27	42.4-51	36.7-44.8	116.9-142.2	94.6-118.5	0.15-0.22
8	0.27-2	56-300	27-50	51-	44.8-250.0	142.2-	118.5-	0.22-0.5
9	2-	300-	50-		250-			0.5-

Table A4 The pollution level of the content of the various potentially toxic trace element grades

Regions	Pollution level	Cd	Pb	As	Ni	Cu	Zn	Cr	Hg
	Clean level	1-7	1-7	1-7	1-5	1-7	1-7	1-7	1-7
SJ	Mixed pollution level	8	8	8	6	8	8,9	8,9	8
	Serious pollution level	9	9	9	7	9			9
	Clean level	1-7	1-8	1-6	1-7	1-7	1-7	1-7	1-8
SN	Mixed pollution level	8	9	7	8,9	8	8,9	8,9	9
	Serious pollution level	9		8		9			
	Clean level	1-7	1-8	1-6	1-7	1-7	1-7	1-7	1-8
HHH	Mixed pollution level	8	9	7	8,9	8	8,9	8,9	9
	Serious pollution level	9		8		9			
	Clean level	1-7	1-7	1-7	1-5	1-7	1-7	1-7	1-7
CJ	Mixed pollution level	8	8	8	6	8	8,9	8,9	8
	Serious pollution level	9	9	9	7	9			9
	Clean level	1-7	1-7	1-7		1-7	1-7	1-7	1-7
SC	Mixed pollution level	8	8	8	6	8	8,9	8,9	8
	Serious pollution level	9	9	9	7	9			9

SJ: Sanjiang Plain; SN: Songnen Plain; CJ: Yangtze River Middle Plain and Jianghuai Plain; HHH: Huang-Huai-Hai Plain; SC: Sichuan Basin.

Table A5 Percentages of class distributions, based on the pollution index (n = 3006), of the assessments of potentially toxic trace element pollution in farmland soils

Class	Cd	Pb	As	Ni	Cu	Zn	Cr	Hg
Five major grain-producing regions (FMGPR)								
Clean	10.60	0.10	1.26	16.65	6.00	3.30	0.64	2.04
Sub-clean	72.02	99.76	97.85	74.94	89.95	93.86	98.73	95.40
Slight pollution	10.70	0.03	0.42	7.69	3.44	1.81	0.48	1.63
Moderate pollution	2.33	0.07	0.09	0.30	0.12	0.23	0.04	0.42
Severe pollution	4.35	0.03	0.37	0.42	0.48	0.79	0.12	0.51
Sanjiang Plain (SJ)								
Clean	5.17	0.00	0.00	26.79	8.77	0.00	0.00	0.00
Sub-clean	93.10	100.00	100.00	73.21	91.23	100.00	100.00	100.00
Slight pollution	0.00	0.00	0.00	0.00	0.00	0.00	0.00	0.00
Moderate pollution	1.72	0.00	0.00	0.00	0.00	0.00	0.00	0.00
Severe pollution	0.00	0.00	0.00	0.00	0.00	0.00	0.00	0.00
Songnen Plain (SN)								
Clean	3.46	0.00	0.00	3.31	0.65	2.03	0.00	0.49

Class	Cd	Pb	As	Ni	Cu	Zn	Cr	Hg
Sub-clean	86.79	100.00	99.53	96.32	99.02	97.64	100.00	99.51
Slight pollution	1.89	0.00	0.47	0.00	0.33	0.34	0.00	0.00
Moderate pollution	4.09	0.00	0.00	0.37	0.00	0.00	0.00	0.00
Severe pollution	3.77	0.00	0.00	0.00	0.00	0.00	0.00	0.00
Huang-Huai-Hai Plain (HHH)								
Clean	4.01	0.00	1.51	4.57	1.66	1.94	0.17	0.68
Sub-clean	85.23	100.00	97.92	93.14	97.52	95.20	98.83	96.51
Slight pollution	4.65	0.00	0.19	1.27	0.83	1.84	0.84	1.84
Moderate pollution	1.28	0.00	0.09	0.25	0.00	0.20	0.00	0.39
Severe pollution	4.82	0.00	0.28	0.76	0.00	0.82	0.17	0.58
Yangtze River Middle Plain and Jianghuai Plain (CJ)								
Clean	13.54	0.28	0.92	27.44	14.57	7.51	1.25	4.67
Sub-clean	64.58	99.59	97.02	56.10	77.15	87.86	98.04	92.29
Slight pollution	15.03	0.00	0.92	16.16	7.21	2.50	0.36	1.40
Moderate pollution	1.64	0.00	0.23	0.00	0.00	0.58	0.18	0.93
Severe pollution	5.21	0.14	0.92	0.30	1.07	1.54	0.18	0.70
Sichuan Basin								
Clean	28.57	0.21	1.89	57.47	7.58	2.33	1.80	3.72
Sub-clean	36.53	99.17	97.30	12.22	83.08	95.00	98.20	93.26
Slight pollution	27.14	0.21	0.54	29.41	7.32	2.33	0.00	2.33
Moderate pollution	4.90	0.41	0.00	0.90	0.76	0.00	0.00	0.23
Severe pollution	2.86	0.00	0.27	0.00	1.26	0.33	0.00	0.47

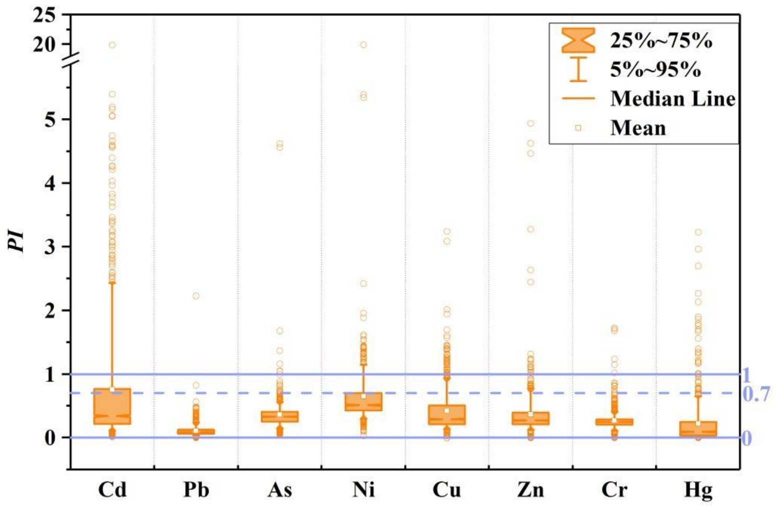

Fig. A1 The pollution index for potentially toxic trace elements (PTEs) in farmland soils in the five major grain-producing regions of China.

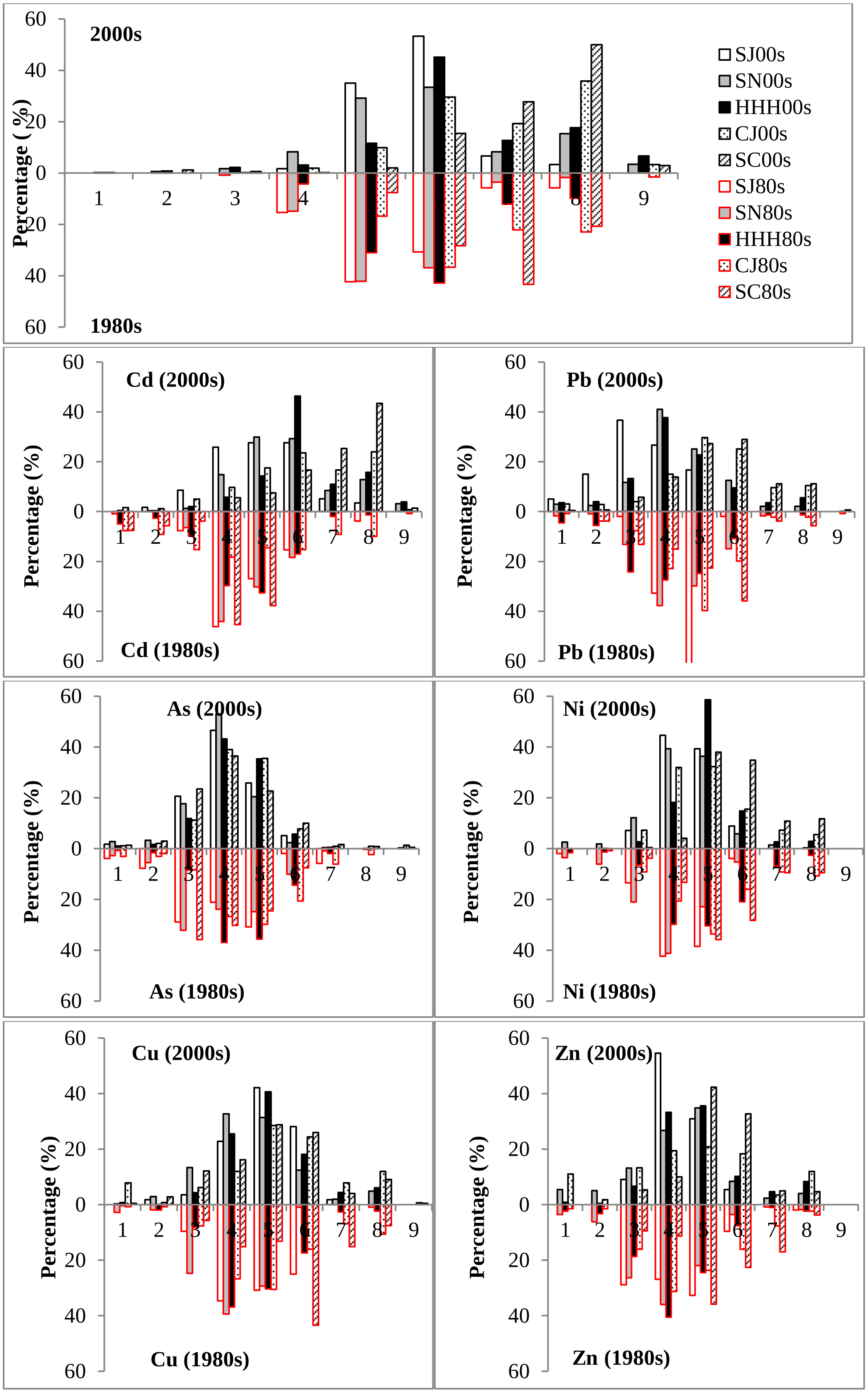
2000s
1980s
Percentage (%)
SJ00s
SN00s
HHH00s
CJ00s
SC00s
SJ80s
SN80s
HHH80s
CJ80s
SC80s
Cd (2000s)
Cd (1980s)
Pb (2000s)
Pb (1980s)
As (2000s)
As (1980s)
Ni (2000s)
Ni (1980s)
Cu (2000s)
Cu (1980s)
Zn (2000s)
Zn (1980s)

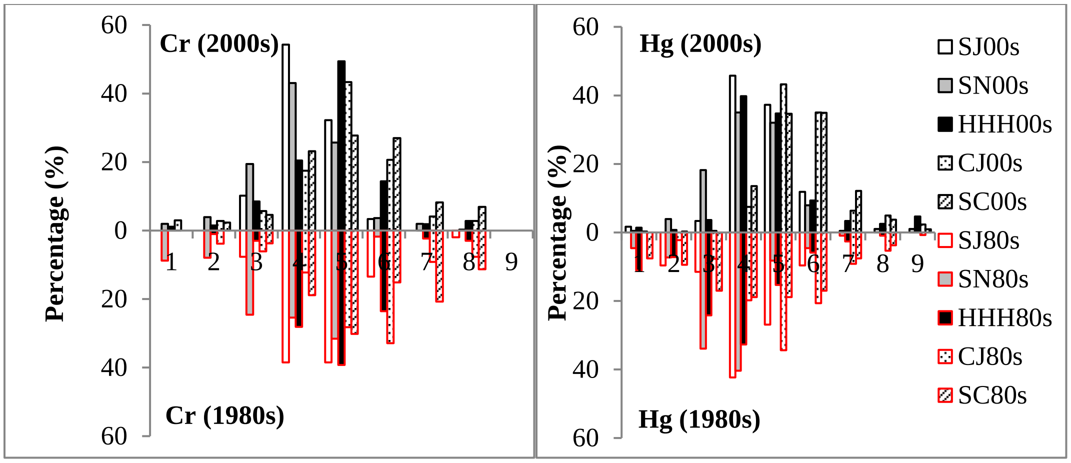

Fig. A2 Trends in the concentrations of potentially toxic trace elements in the cultivated lands of the five major grain-producing regions in China from the 1980s to 2000s

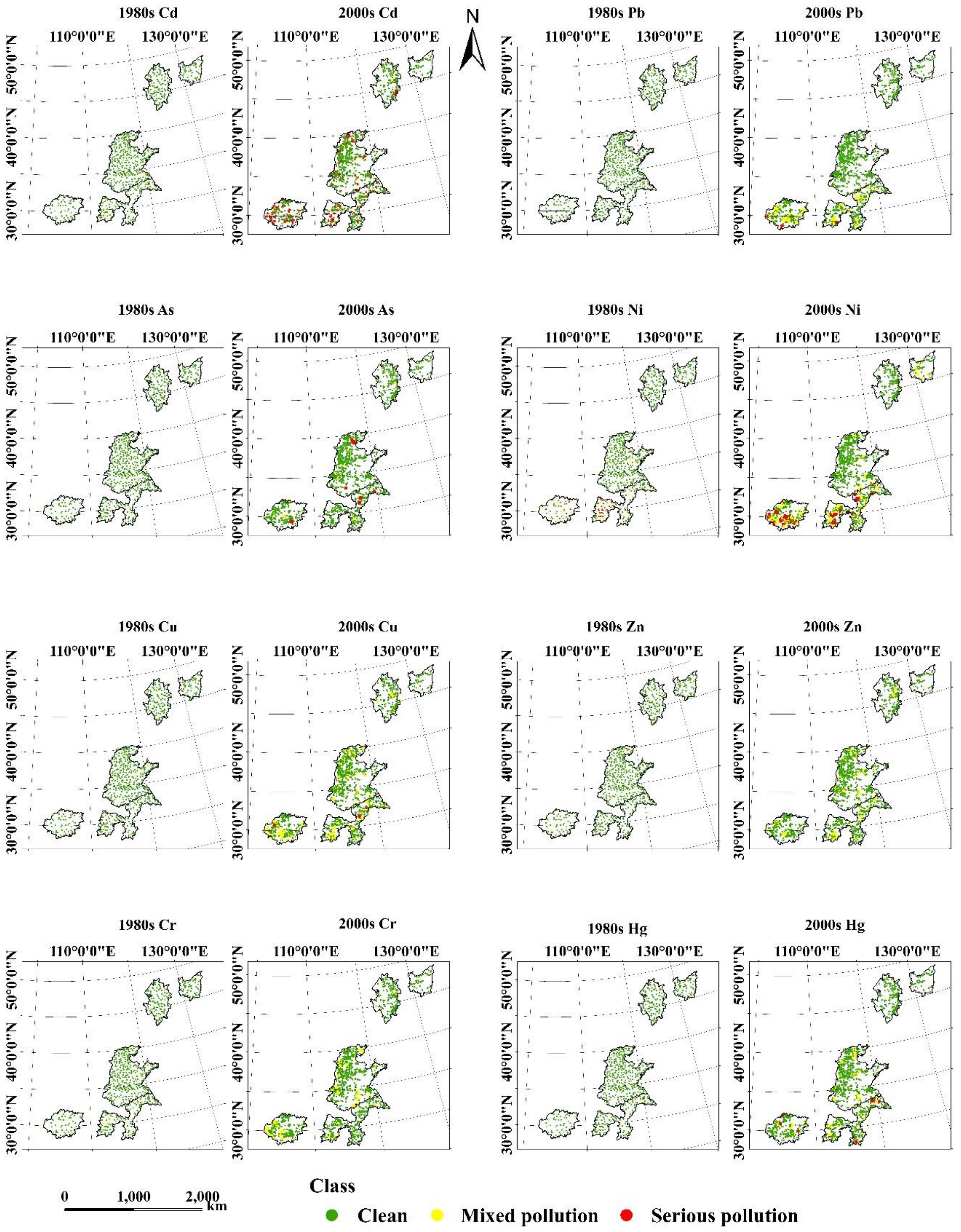

Fig. A3 A comparison map of the spatial distribution of different potentially toxic trace element pollution levels in the cultivated lands of the five major grain-producing regions in China in the two decades (the 1980s and 2000s) at point-scale.
